# 6-Chloro-*N*-(pyridin-4-ylmeth­yl)pyridine-3-sulfonamide

**DOI:** 10.1107/S1600536813030523

**Published:** 2013-11-13

**Authors:** Parameshwar Adimoole Suchetan, Revanasiddappa Nadigar Mohan, Vijithkumar, Bandrehalli Siddagangaiah Palakshamurthy, Swamy Sreenivasa

**Affiliations:** a Department of Studies and Research in Chemistry, U.C.S., Tumkur University, Tumkur Karnataka 572 103, India; b Soild State and Structural Chemistry Unit, Indian Institute of Science, Bangalore, India; c Department of Studies and Research in Physics, U.C.S., Tumkur University, Tumkur Karnataka 572 103, India

## Abstract

In the title sulfonamide derivative, C_11_H_10_ClN_3_O_2_S, the dihedral angle between the pyridine rings is 46.85 (12)°. The N atom of the chloro­pyridine ring is *anti* to the N—H bond. In the crystal, mol­ecules are linked through N—H⋯N hydrogen bonds into zigzag chains parallel to [001] with a *C*(7) graph-set motif.

## Related literature

For graph-set analysis of hydrogen-bond patterns, see: Bernstein *et al.* (1995 ▶). For the anti­microbial activity of related compounds, see: Desai *et al.* (2013 ▶); Mohan *et al.* (2013 ▶). For the proliferation activity of these compounds, see: Renu *et al.* (2006 ▶), and for their tuberculostaic acitivity, see: Gobis *et al.* (2013 ▶).
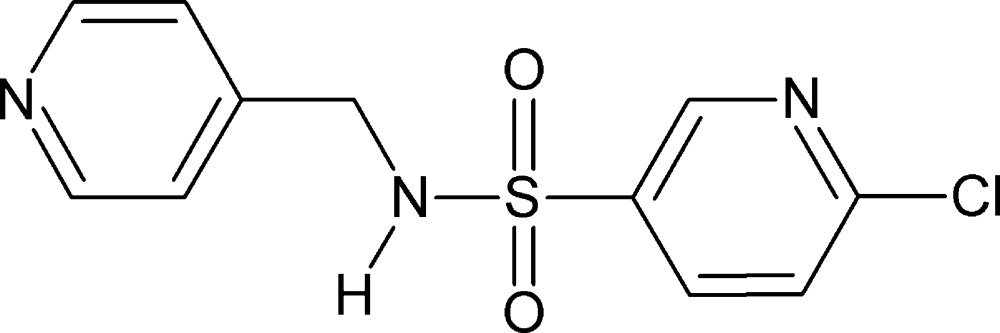


## Experimental

### 

#### Crystal data

C_11_H_10_ClN_3_O_2_S
*M* *_r_* = 283.73
Monoclinic, 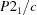

*a* = 5.4140 (6) Å
*b* = 18.172 (2) Å
*c* = 12.9392 (15) Å
β = 92.388 (6)°
*V* = 1271.9 (2) Å^3^
*Z* = 4
Mo *K*α radiation
μ = 0.46 mm^−1^
*T* = 293 K
0.35 × 0.29 × 0.23 mm

#### Data collection

Bruker APEXII CCD diffractometer
Absorption correction: multi-scan (*SADABS*; Bruker, 2009 ▶) *T*_min_ = 0.852, *T*_max_ = 0.899
20929 measured reflections
2961 independent reflections
2185 reflections with *I* > 2σ(*I*)
*R*_int_ = 0.035

#### Refinement

*R*[*F* ^2^ > 2σ(*F* ^2^)] = 0.044
*wR*(*F* ^2^) = 0.137
*S* = 0.82
2961 reflections
167 parameters
H atoms treated by a mixture of independent and constrained refinement
Δρ_max_ = 0.27 e Å^−3^
Δρ_min_ = −0.39 e Å^−3^

### 

Data collection: *APEX2* (Bruker, 2009 ▶); cell refinement: *SAINT-Plus* (Bruker, 2009 ▶); data reduction: *SAINT-Plus*; program(s) used to solve structure: *SHELXS97* (Sheldrick, 2008 ▶); program(s) used to refine structure: *SHELXL97* (Sheldrick, 2008 ▶); molecular graphics: *Mercury* (Macrae *et al.*, 2008 ▶); software used to prepare material for publication: *SHELXL97*.

## Supplementary Material

Crystal structure: contains datablock(s) I, New_Global_Publ_Block. DOI: 10.1107/S1600536813030523/wm2782sup1.cif

Structure factors: contains datablock(s) I. DOI: 10.1107/S1600536813030523/wm2782Isup2.hkl

Supplementary material file. DOI: 10.1107/S1600536813030523/wm2782Isup3.cml

Additional supplementary materials:  crystallographic information; 3D view; checkCIF report

## Figures and Tables

**Table 1:** Hydrogen-bond geometry (Å, °)

*D*—H⋯*A*	*D*—H	H⋯*A*	*D*⋯*A*	*D*—H⋯*A*
N2—H*N*2⋯N3^i^	0.78 (3)	2.10 (3)	2.870 (3)	174.53

Symmetry code: (i) 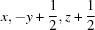.

**Fig. 1.**
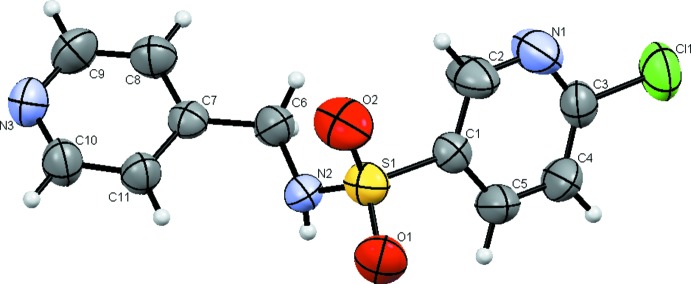
Molecular structure of the title compound, showing the atom-labelling scheme. Displacement ellipsoids are drawn at the 50% probability level.

**Fig. 2.**
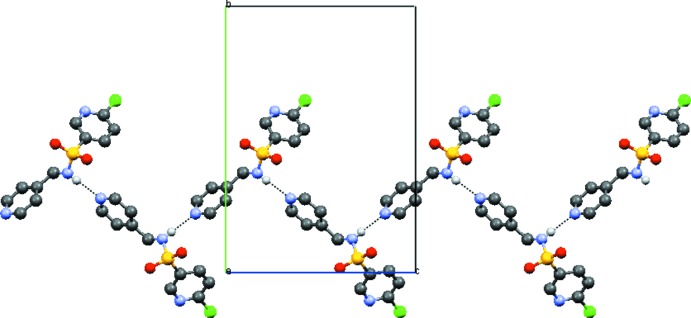
Linking of individual molecules into C(7) chains parallel to [001] through N—H···N hydrogen bonds. H-atoms not involved in H-bonding are omitted for clarity.

## supplementary crystallographic information

### 1. Comment

Pyridine ring containing sulfonamide moieties show antimicrobial activity (Desai *et al.*, 2013; Mohan *et al.*, 2013), proliferation activity (Renu *et al.*, 2006) and tuberculostaic acitivity (Gobis *et al.*, 2013). Keeping this in mind, the title compound, C_11_H_10_ClN_3_O_2_S, (I), was synthesized and its crystal structure determined.

In the structure of compound (I) the dihedral angle between the two pyridine rings is 46.85(12°. The N-atom of the chloropyridine ring in the compound is anti to the N—H bond (Fig 1). In the crystal structure, the molecules are linked through N2—H*N*2···N3 hydrogen bonds (Table 1, Fig. 2) into zigzag chains with graph-set notation *C*(7) (Bernstein *et al.* 1995) running parallel to [001] .

### 2. Experimental

Pyridin-4-ylmethanamine (7.4 mmol) was taken in dry dichloromethane (10 ml) and cooled to 273 K. To this solution 6-chloropyridine-3-sulfonyl chloride (7.4 mmol) in dichloromethane and triethylamine (1.48 mmol) was added slowly and the solution was heated to 323 K for 4 h. The reaction was monitored by TLC. The reaction mixture was cooled and washed with 10% sodium bicarbonate solution. The organic layer was separated, dried and concentrated to obtain the crude compound. It was purified by column chromatography using petroleum ether: ethyl acetate (7:3) as eluent.

Yellow prisms of the title compound suitable for diffraction studies were obtained from evapouration of the solution of the compound in a mixture of petroleum ether: ethyl acetate (7:3).

### 3. Refinement

The H atom of the NH group was located in a difference map and refined freely. The other H atoms were positioned with idealized geometry using a riding model with C—H = 0.93 Å (aromatic) and 0.97 Å (methylene). Isotropic displacement parameters for all H atoms were set to 1.2 times *U*_eq_ of the parent atom.

### Crystal data

**Table:**

C_11_H_10_ClN_3_O_2_S	Prism
*M**_r_* = 283.73	*D*_x_ = 1.482 Mg m^−^^3^
Monoclinic, *P*2_1_/*c*	Melting point: 492 K
Hall symbol: -P 2ybc	Mo *K*α radiation, λ = 0.71073 Å
*a* = 5.4140 (6) Å	Cell parameters from 1103 reflections
*b* = 18.172 (2) Å	θ = 1.9–27.8°
*c* = 12.9392 (15) Å	µ = 0.46 mm^−^^1^
β = 92.388 (6)°	*T* = 293 K
*V* = 1271.9 (2) Å^3^	Prism, yellow
*Z* = 4	0.35 × 0.29 × 0.23 mm
*F*(000) = 584	

### Data collection

**Table:**

Bruker APEXII CCD diffractometer	2961 independent reflections
Radiation source: fine-focus sealed tube	2185 reflections with *I* > 2σ(*I*)
Graphite monochromator	*R*_int_ = 0.035
φ and ω scans	θ_max_ = 27.8°, θ_min_ = 1.9°
Absorption correction: multi-scan (*SADABS*; Bruker, 2009)	*h* = −7→7
*T*_min_ = 0.852, *T*_max_ = 0.899	*k* = −23→23
20929 measured reflections	*l* = −13→16

### Refinement

**Table:**

Refinement on *F*^2^	Primary atom site location: structure-invariant direct methods
Least-squares matrix: full	Secondary atom site location: difference Fourier map
*R*[*F*^2^ > 2σ(*F*^2^)] = 0.044	Hydrogen site location: inferred from neighbouring sites
*wR*(*F*^2^) = 0.137	H atoms treated by a mixture of independent and constrained refinement
*S* = 0.82	*w* = 1/[σ^2^(*F*_o_^2^) + (0.0847*P*)^2^ + 0.7427*P*] where *P* = (*F*_o_^2^ + 2*F*_c_^2^)/3
2961 reflections	(Δ/σ)_max_ < 0.001
167 parameters	Δρ_max_ = 0.27 e Å^−^^3^
0 restraints	Δρ_min_ = −0.39 e Å^−^^3^

### Special details

**Table:**

Geometry. All s.u.'s (except the s.u. in the dihedral angle between two l.s. planes) are estimated using the full covariance matrix. The cell s.u.'s are taken into account individually in the estimation of s.u.'s in distances, angles and torsion angles; correlations between s.u.'s in cell parameters are only used when they are defined by crystal symmetry. An approximate (isotropic) treatment of cell s.u.'s is used for estimating s.u.'s involving l.s. planes.
Refinement. Refinement of *F*^2^ against ALL reflections. The weighted *R*-factor *wR* and goodness of fit *S* are based on *F*^2^, conventional *R*-factors *R* are based on *F*, with *F* set to zero for negative *F*^2^. The threshold expression of *F*^2^ > 2σ(*F*^2^) is used only for calculating *R*-factors(gt) *etc*. and is not relevant to the choice of reflections for refinement. *R*-factors based on *F*^2^ are statistically about twice as large as those based on *F*, and *R*- factors based on ALL data will be even larger.

### Fractional atomic coordinates and isotropic or equivalent isotropic displacement parameters (Å^2^)

**Table:**

	*x*	*y*	*z*	*U*_iso_*/*U*_eq_	
HN2	0.486 (4)	0.3469 (13)	0.213 (2)	0.058 (7)*	
S1	0.32655 (10)	0.44681 (3)	0.19440 (4)	0.0590 (2)	
Cl1	1.10225 (15)	0.64661 (4)	0.41805 (6)	0.0911 (3)	
C1	0.5418 (4)	0.50525 (11)	0.25986 (16)	0.0486 (4)	
C7	0.6225 (4)	0.32076 (10)	0.00501 (15)	0.0474 (4)	
N2	0.4712 (4)	0.37356 (11)	0.16614 (15)	0.0584 (5)	
C3	0.8769 (4)	0.59143 (12)	0.35659 (18)	0.0600 (5)	
N3	0.5621 (4)	0.22140 (11)	−0.15918 (16)	0.0692 (5)	
C11	0.4300 (4)	0.27124 (12)	−0.00074 (17)	0.0571 (5)	
H11	0.3157	0.2700	0.0509	0.068*	
N1	0.8263 (5)	0.60509 (13)	0.2581 (2)	0.0899 (7)	
O1	0.1498 (3)	0.42543 (11)	0.26714 (16)	0.0804 (5)	
O2	0.2501 (4)	0.48324 (12)	0.10098 (16)	0.0912 (6)	
C10	0.4071 (4)	0.22353 (13)	−0.08329 (19)	0.0653 (6)	
H10	0.2746	0.1909	−0.0856	0.078*	
C5	0.6049 (6)	0.49344 (15)	0.36172 (19)	0.0785 (8)	
H5	0.5314	0.4553	0.3973	0.094*	
C2	0.6559 (6)	0.56102 (15)	0.2099 (2)	0.0822 (8)	
H2	0.6153	0.5691	0.1402	0.099*	
C8	0.7862 (5)	0.31869 (14)	−0.0734 (2)	0.0676 (6)	
H8	0.9200	0.3508	−0.0731	0.081*	
C6	0.6641 (4)	0.37509 (14)	0.09095 (17)	0.0635 (6)	
H6A	0.8220	0.3648	0.1262	0.076*	
H6B	0.6725	0.4241	0.0617	0.076*	
C4	0.7741 (6)	0.53707 (15)	0.41083 (18)	0.0797 (8)	
H4	0.8183	0.5298	0.4803	0.096*	
C9	0.7497 (5)	0.26847 (16)	−0.1525 (2)	0.0805 (8)	
H9	0.8635	0.2675	−0.2044	0.097*	

### Atomic displacement parameters (Å^2^)

**Table:**

	*U*^11^	*U*^22^	*U*^33^	*U*^12^	*U*^13^	*U*^23^
S1	0.0487 (3)	0.0671 (4)	0.0603 (4)	−0.0063 (2)	−0.0084 (2)	0.0061 (3)
Cl1	0.0924 (5)	0.0868 (5)	0.0934 (5)	−0.0227 (4)	−0.0072 (4)	−0.0295 (4)
C1	0.0463 (10)	0.0461 (10)	0.0534 (11)	0.0038 (8)	0.0016 (8)	0.0003 (8)
C7	0.0480 (10)	0.0485 (10)	0.0454 (10)	0.0020 (8)	−0.0032 (8)	0.0064 (8)
N2	0.0767 (12)	0.0571 (10)	0.0413 (10)	−0.0150 (9)	0.0034 (8)	0.0012 (8)
C3	0.0623 (13)	0.0550 (12)	0.0626 (14)	−0.0032 (10)	0.0027 (10)	−0.0153 (10)
N3	0.0808 (13)	0.0633 (11)	0.0640 (12)	0.0020 (10)	0.0095 (10)	−0.0132 (9)
C11	0.0542 (11)	0.0622 (12)	0.0554 (12)	−0.0072 (10)	0.0088 (9)	−0.0077 (10)
N1	0.1045 (18)	0.0783 (14)	0.0853 (17)	−0.0339 (13)	−0.0122 (14)	0.0155 (12)
O1	0.0526 (9)	0.0965 (13)	0.0928 (13)	−0.0144 (9)	0.0127 (9)	−0.0050 (11)
O2	0.0814 (12)	0.1002 (14)	0.0883 (14)	−0.0074 (10)	−0.0385 (11)	0.0283 (11)
C10	0.0620 (13)	0.0641 (13)	0.0700 (15)	−0.0071 (11)	0.0051 (11)	−0.0144 (11)
C5	0.106 (2)	0.0773 (16)	0.0522 (14)	−0.0350 (15)	0.0037 (13)	0.0053 (12)
C2	0.098 (2)	0.0758 (16)	0.0713 (16)	−0.0242 (14)	−0.0187 (15)	0.0251 (13)
C8	0.0635 (14)	0.0694 (14)	0.0710 (15)	−0.0096 (11)	0.0158 (12)	−0.0021 (12)
C6	0.0645 (13)	0.0728 (14)	0.0532 (12)	−0.0219 (11)	0.0026 (10)	−0.0049 (11)
C4	0.111 (2)	0.0838 (17)	0.0432 (12)	−0.0313 (15)	−0.0079 (13)	−0.0001 (11)
C9	0.0896 (19)	0.0863 (18)	0.0678 (16)	−0.0030 (15)	0.0309 (14)	−0.0107 (14)

### Geometric parameters (Å, º)

**Table:**

S1—O1	1.4238 (19)	N3—C9	1.328 (3)
S1—O2	1.4244 (18)	C11—C10	1.377 (3)
S1—N2	1.595 (2)	C11—H11	0.9300
S1—C1	1.767 (2)	N1—C2	1.354 (3)
Cl1—C3	1.745 (2)	C10—H10	0.9300
C1—C2	1.364 (3)	C5—C4	1.350 (3)
C1—C5	1.365 (3)	C5—H5	0.9300
C7—C11	1.376 (3)	C2—H2	0.9300
C7—C8	1.375 (3)	C8—C9	1.379 (4)
C7—C6	1.497 (3)	C8—H8	0.9300
N2—C6	1.457 (3)	C6—H6A	0.9700
N2—HN2	0.78 (3)	C6—H6B	0.9700
C3—N1	1.316 (3)	C4—H4	0.9300
C3—C4	1.346 (3)	C9—H9	0.9300
N3—C10	1.318 (3)		
			
O1—S1—O2	120.54 (13)	N3—C10—H10	118.1
O1—S1—N2	105.88 (11)	C11—C10—H10	118.1
O2—S1—N2	108.75 (13)	C4—C5—C1	120.0 (2)
O1—S1—C1	107.16 (11)	C4—C5—H5	120.0
O2—S1—C1	106.87 (11)	C1—C5—H5	120.0
N2—S1—C1	106.94 (10)	N1—C2—C1	122.3 (2)
C2—C1—C5	118.3 (2)	N1—C2—H2	118.9
C2—C1—S1	121.47 (18)	C1—C2—H2	118.9
C5—C1—S1	120.14 (17)	C7—C8—C9	119.2 (2)
C11—C7—C8	116.9 (2)	C7—C8—H8	120.4
C11—C7—C6	124.15 (19)	C9—C8—H8	120.4
C8—C7—C6	118.97 (19)	N2—C6—C7	113.18 (17)
C6—N2—S1	120.65 (17)	N2—C6—H6A	108.9
C6—N2—HN2	118.6 (19)	C7—C6—H6A	108.9
S1—N2—HN2	112.0 (18)	N2—C6—H6B	108.9
N1—C3—C4	124.7 (2)	C7—C6—H6B	108.9
N1—C3—Cl1	116.52 (19)	H6A—C6—H6B	107.8
C4—C3—Cl1	118.74 (19)	C3—C4—C5	118.2 (2)
C10—N3—C9	116.2 (2)	C3—C4—H4	120.9
C7—C11—C10	119.8 (2)	C5—C4—H4	120.9
C7—C11—H11	120.1	N3—C9—C8	124.1 (2)
C10—C11—H11	120.1	N3—C9—H9	118.0
C3—N1—C2	116.4 (2)	C8—C9—H9	118.0
N3—C10—C11	123.8 (2)		
			
O1—S1—C1—C2	−148.2 (2)	C2—C1—C5—C4	0.9 (4)
O2—S1—C1—C2	−17.7 (3)	S1—C1—C5—C4	178.3 (2)
N2—S1—C1—C2	98.7 (2)	C3—N1—C2—C1	0.0 (5)
O1—S1—C1—C5	34.4 (2)	C5—C1—C2—N1	−0.7 (5)
O2—S1—C1—C5	164.9 (2)	S1—C1—C2—N1	−178.2 (2)
N2—S1—C1—C5	−78.7 (2)	C11—C7—C8—C9	0.3 (3)
O1—S1—N2—C6	178.59 (17)	C6—C7—C8—C9	179.6 (2)
O2—S1—N2—C6	47.7 (2)	S1—N2—C6—C7	−125.57 (19)
C1—S1—N2—C6	−67.37 (19)	C11—C7—C6—N2	−3.3 (3)
C8—C7—C11—C10	−0.9 (3)	C8—C7—C6—N2	177.4 (2)
C6—C7—C11—C10	179.8 (2)	N1—C3—C4—C5	−0.6 (5)
C4—C3—N1—C2	0.7 (5)	Cl1—C3—C4—C5	−178.2 (2)
Cl1—C3—N1—C2	178.4 (2)	C1—C5—C4—C3	−0.2 (5)
C9—N3—C10—C11	0.5 (4)	C10—N3—C9—C8	−1.2 (4)
C7—C11—C10—N3	0.5 (4)	C7—C8—C9—N3	0.8 (4)

### Hydrogen-bond geometry (Å, º)

**Table:**

*D*—H···*A*	*D*—H	H···*A*	*D*···*A*	*D*—H···*A*
N2—H*N*2···N3^i^	0.78 (3)	2.10 (3)	2.870 (3)	174.53

Symmetry code: (i) *x*, −*y*+1/2, *z*+1/2.
